# Oral Absorbent AST-120 Is Associated with Compositional and Functional Adaptations of Gut Microbiota and Modification of Serum Short and Medium-Chain Fatty Acids in Advanced CKD Patients

**DOI:** 10.3390/biomedicines10092234

**Published:** 2022-09-08

**Authors:** Cheng-Kai Hsu, Shih-Chi Su, Lun-Ching Chang, Kai-Jie Yang, Chin-Chan Lee, Heng-Jung Hsu, Yih-Ting Chen, Chiao-Yin Sun, I-Wen Wu

**Affiliations:** 1 Department of Nephrology, Chang Gung Memorial Hospital, Keelung 204, Taiwan; 2 Whole-Genome Research Core Laboratory of Human Diseases, Chang Gung Memorial Hospital, Keelung 204, Taiwan; 3 Department of Mathematical Sciences, Florida Atlantic University, Boca Raton, FL 33431, USA; 4 College of Medicine, Chang Gung University, Taoyuan 333, Taiwan

**Keywords:** AST-120, chronic kidney disease, gut microbiota, kidney health, short-chain fatty acids

## Abstract

Background: Animal studies have demonstrated that an oral absorbent AST-120 modulates gut environment. However, this phenomenon remains unclear in humans. This study aimed to assess the effects of AST-120 on the gut microbiota, related functional capability and metabolomic profiling in advanced chronic kidney diseases (CKD) patients. Methods: Eight advanced CKD patients with AST-120 (CKD+AST), 24 CKD patients (CKD), and 24 non-CKD controls were enrolled. We analyzed 16S rRNA pyrosequencing of feces and serum metabolomics profiling. Results: The CKD+AST group exhibited dispersed microbial community structure (β-diversity, *p* < 0.001) compared to other groups. The relative abundances of at least 16 genera were significantly different amongst the three groups. Increases of fatty acids-producing bacteria (*Clostridium_sensu_stricto_1*, *Ruminococcus_2*, *Eubacterium_nodatum* and *Phascolarctobacterium*) associated with elevated serum acetic acid and octanoic acid levels were found in CKD+AST group. Analysis of microbial gene function indicated that pathway modules relevant to metabolisms of lipids, amino acids and carbohydrates were differentially enriched between CKD+AST and CKD groups. Specifically, enrichments of gene markers of the biosynthesis of fatty acids were noted in the CKD+AST group. Conclusion: Advanced CKD patients exhibited significant gut dysbiosis. AST-120 can partially restore the gut microbiota and intervenes in a possible signature of short- and medium-chain fatty acids metabolism.

## 1. Introduction

The gut dysbiosis associated with uremic milieu produces toxic metabolites. The impaired renal function can further lead to accumulation of uremic toxins. These solutes reach the intestine, cause alterations in bacteria composition and exacerbate fecal metabolite profile, inducing a positive feedback that allows translocation of endotoxins into the bloodstream, which enhances systemic and kidney inflammation and further aggravates kidney injury [1,2]. Indoxyl sulfate (IS) and *p*-cresyl sulfate (pCS) are representative uremic toxins that can increase oxidative stress and contribute to systemic inflammation, cardiovascular disease, renal damage and mortality in chronic kidney disease (CKD) patients [3,4,5,6]. An orally administered spherical carbon absorbent AST-120, which has been commercialized since 1991 in Japan and then became worldwide, has the potential of renal protection and cardiovascular benefits in patients with progressive CKD via absorbing the IS and pCS precursors generated by amino acid metabolism within the gastrointestinal tract [7,8,9,10,11]. In addition to the absorption of the uremic toxin precursors, animal studies have shown that AST-120 may affect the composition of intestinal microbiota, thereby influencing metabolomic profiling [12,13]. Furthermore, AST-120 was associated with attenuated damage to the epithelial tight junction of the colon, which was disrupted during CKD progression [14]. Although significant changes of composition of microbiota were found on the murine gut following administration of AST-120, this evidence remains largely incomplete in human experiments.

The perspective of the bidirectional relationship between gut microbiota and kidney disease is still evolving [15]. CKD is associated with specific changes in gut microbiota and circulating metabolome. Recent studies showed usefulness of specific gut metagenomic and metabolomic markers to discriminate different severities of CKD in clinical settings [16,17]. Additionally, there were modifications of gut microbiota and related metabolites associated with dietary patterns, such as low protein diet, in CKD patients [18]. Nevertheless, modifications of intestinal microbiota and related metabolites associated with administration of AST-120 in advanced CKD patients remain unclear. Thus, this study aims to assess the effects of AST-120 on the gut microbiota and related metabolomic profiling in patients with advanced CKD.

## 2. Materials and Methods

### 2.1. Study Design and Patient Recruitment

Thirty-two advanced CKD patients (stage 4–5) and twenty-four non-CKD controls were enrolled. We matched participants according to age, gender, diabetes and hypertension. All patients were recruited from Chang Gung Memorial Hospital, Keelung, Taiwan. Advanced CKD was defined if estimated glomerular filtration rate (eGFR, Modification of Diet in Renal Disease equation) of less than 30 mL/min/1.73 m^2^ in two separate occasions. For those with advanced CKD, 8 patients were treated with AST-120 for 3 months (6 g/day, CKD+AST group) and 24 patients did not receive AST-120 (CKD group). We excluded those who receiving renal replacement therapy, kidney transplantation, cardiovascular disease, malignancy, liver cirrhosis, active infection, previously abdominal surgery, previous use of AST-120, or use of probiotics, prebiotics or antibiotics at enrollment. The participants were not permitted to take any supplement containing probiotics, such as yogurt, within 7 days before sample collection. All fasting fresh stools and plasma were appropriately collected at the end of the third month and stored at −80 °C until analysis. A minimal of 54 total samples was calculated to have a study power of 0.9 and α-error probability of 0.05 in a three-group design (non-CKD control, CKD, and CKD+AST groups), based on effect size of 50% and significance level at 0.05 under two-tail analysis. A total number of 56 patients were justified by sample size calculation statement. The Institutional Review Board at Chang Gung Memorial Hospital (IRB: 202002535B0, 201900167B0, 201800273B0C602) approved this study. The written consents were collected from study participants (Clinical Trial gov. NCT04300387).

### 2.2. Gut-Producing Metabolites

Detailed methodology of metabolomics was illustrated in previous studies [17,18,19]. Briefly, we investigated serum levels of short-chain fatty acids (SCFA) and medium-chain fatty acids (MCFA, Table S1) by GC-MS analysis. Agilent 7890B gas chromatograph system coupled with an Agilent 5977B mass spectrometer were used. We detected 41 circulating bile acids (Table S2), by UHPLC-MS/MS analysis. UHPLC separation was performed in an Agilent 1290 Infinity series UHPLC system, equipped with a Waters ACQUITY UPLC BEH C18 column (150 × 2.1 mm, 1.7 μm, Waters, Agilent Technologies, Santa Clara, CA, USA). Q Exactive Focus mass spectrometer (Thermo Fisher Scientific, Waltham, MA, USA) was used to estimate the MS analysis. Circulating pCS and IS (free and protein-bound fractions) were analyzed with UPLC-MS/MS (Milford, MA, USA). Concentrations of free pCS and IS were measured in serum ultrafiltrates by using AmicoUltra 30 K filter (Millipore, Burlington, MA, USA). Samples were deproteinized by the addition of acetonitrile. Chromatographic separation was performed at 30 °C using an ACQUITY UPLC BEH C18 column (2.1 × 100 nm). The analytes were quantified with Waters ACQUITY UPLC Xevo TQ-S operating in negative electrospray ionization and multiple reaction monitoring mode [16,19].

### 2.3. Fecal 16S rRNA Gene Sequencing and Functional Prediction of Bacterial Gene

We extracted fecal bacterial DNA by the FastDNA SPIN Kit. The 16S rRNA gene sequencing, data processing and analysis were described in our previous works [16,18]. In brief, we conducted a polymerase chain reaction (PCR) to amplify the variable region 4 (V4) of the gene encoding 16S rRNA in microbiota. Additionally, sequencing was carried out on an Illumina HiSeq 2500 platform. Sequencing reads (effective tags) were grouped into operational taxonomic units (OTU) at 97% sequence identity using UPARSE [20]. We used the SILVA database to define bacterial taxonomy classification [21]. We determined α and β-diversity by means of Chao1 index and Bray–Curtis dissimilarities, respectively. Non-metric dimensional scaling (NMDS) was calculated in R software. We dissected the functional composition of metagenomes predicted from 16S rRNA data via phylogenetic reconstruction of unobserved states (PICRUSt) software [22]. In addition, we checked gene content prediction based on the IMG database [23] and phylogenetic tree by the Greengenes database [24].

### 2.4. Statistical Analysis

Categorical variables were presented as frequency (percentage) and compared using Fisher’s exact test. Continuous variables of clinical indices were expressed as means ± standard deviation (SD) or median (interquartile range, IQR) and compared using the Student *t*-test or Kruskal–Wallis test. The Kolmogorov–Simirnov method was used to test the normality of numerical variables. We applied the Kruskal–Wallis test to calculate the Chao1 index; and we used the Wilcoxon rank sum test to estimate the Bray–Curtis distance between groups. The compositional discrimination between groups was further evaluated by analysis of similarities (ANOSIM) of UniFrac parameters using 999 permutations in each test. Significant differences regarding the relative abundance of OTU among the three groups were compared (genus level) by the Kruskal–Wallis test. Further post-hoc analyses between two groups were conducted by using Dunn’s test. We further evaluated statistically significant taxa by linear discriminant analysis (LDA) of effect size (LEfSe) analysis. The non-parametric factorial Kruskal–Wallis test, Wilcoxon rank sum test and LDA were employed to identify differentially abundant taxa between two metadata classes. The student *t*-test was conducted to analyze the differences in abundance of genes regarding metabolism pathways between groups. Statistical analysis was performed with the Statistical Package for the Social Sciences version 21.0 (SPSS, Inc., Chicago, IL, USA). All statistical tests were two-tailed, and a *p*-value < 0.05 was considered statistically significant.

## 3. Results

### 3.1. Subject Characteristics

The baseline characteristics of three groups (n = 56) were shown in Table 1. The mean age of subjects was 66.3 ± 7.7 years, and 24 (42.9%) were men. There were no statistically significant differences between CKD and CKD+AST groups regarding age, gender, diabetes, hypertension, gout, hyperlipidemia, blood pressure, body mass index, renal functions, and electrolytes.

### 3.2. Modification of Gut Microbial Architecture in Advanced CKD Patients Receiving AST-120

**Figure 1:**
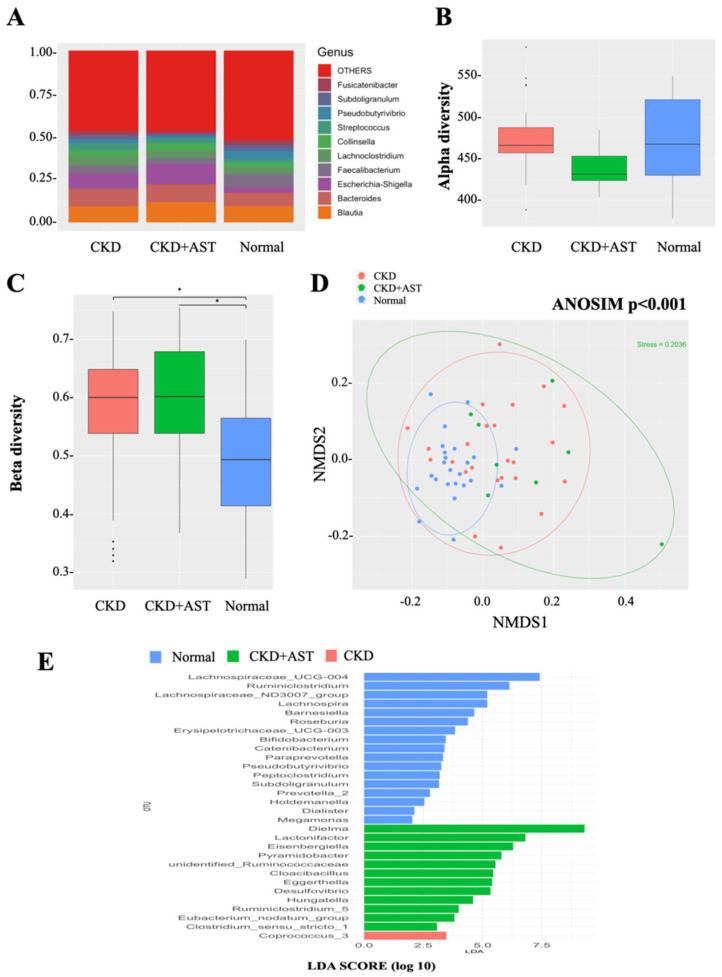
Differences in intestinal microbiota composition and diversity in normal renal function controls and advanced CKD patients receiving AST-120 or not. (**A**) Top 10 genera among groups. The relative abundances are expressed as percentage. “1” denotes 100%. (**B**) α-diversity (Chao 1) and (**C**) β-diversity (Bray–Curtis similarity index) of intestinal microbiota among groups. The box-plot reveals the median, the 25th, and the 75th percentile; * *p* < 0.001. (**D**) Nonmetric multidimensional scaling (NMDS) ordination based on weighted UniFrac of gut microbiomes. Significant sample-to-sample dissimilarities by analysis of similarity (ANOSIM, *p* < 0.001) test for determining differences in microbial composition among three groups. (**E**) Microbial taxa that best characterize each group were defined by applying linear discriminant analysis of effect size (LEfSe) on OTU tables.

To characterize the gut microbial composition between the three groups, we conducted a taxonomic analysis of top-10 genus and detected a predominance of Escherichia-Shigella (11.9%, i.e., 11.9% of the overall sequenced reads) in the CKD+AST group as compared to the CKD group (8.9%) and normal controls (3.5%, *p* = 0.002). Significant reductions of Fusicatenibacter (1.5% vs. 2.5% vs. 2.9 %, *p* = 0.029), Subdoligranulum (1.3% vs. 2.6% vs. 3.3%, *p* = 0.005) and Faecalibacterium (3.3% vs. 4.4% vs. 7.5%, *p* = 0.003) were observed in the CKD+AST group compared to the CKD group and normal controls, respectively. The Figure 1A displays the taxonomic differences (at the genus level) among the three groups (Figure 1A). A moderate but not significant decrease in α-diversity was noted in the CKD+AST group as compared to the CKD group and to the subjects with normal renal function (Figure 1B). On the other hand, analyses of sample-to-sample dissimilarities in microbial communities revealed that β-diversity of gut microbiome in CKD and CKD+AST group was higher than that in normal controls (Figure 1C). Advanced CKD patients receiving AST-120 exhibited a highly dispersed microbial community structure in term of between-sample differences, as compared to the other two study groups (Figure 1D, ANOSIM, *p* < 0.001).

Because significant changes of composition of gut microbiome were observed among the groups, we further explored the changes of relative abundances of specific intestinal microorganisms at the genus level. Significant differences of the relative abundances of 16 genera were identified among the three groups using strict criteria (genus present in >90% of sample and relative abundances > 0.1% in each sample). Specifically, we found that CKD+AST group harbored increased levels of *Bilophila*, *Desulfovibrio*, *Cloacibacillus*, *Pyramidobacter*, *Hungatella*, *Clostridium sensu stricto_1*, *Phascolarctobacterium*, *Eggerthella*, *Eubacterium nodatum* and *Ruminococcus_2*, and, reductions of relative abundances of *Erysipelotrichaceae_UCG-003*, *Catenibacterium*, *Coprococcus_3*, *Lachnospira*, *Prevotella_9* and *Barnesiella* compared to the CKD group (Table 2). In addition, further analyses were performed to display OTUs associated with AST-120 treatment. We predicted the biomarkers for CKD+AST vs. CKD group by taking statistical significance and biological consistency into consideration using LEfSe. Accordingly, among possible markers identified, CKD+AST group consistently exhibited substantial enrichments for *Desulfovibrio*, *Cloacibacillus*, *Pyramidobacter*, *Hungatella*, *Clostridium_sensu_stricto_1* (Figure 1E).

### 3.3. Description of Variation of Metabolites in Advanced CKD Patients Receiving AST-120

**Figure 2:**
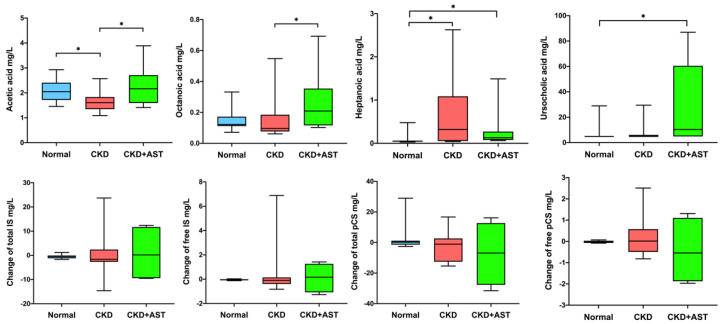
Changes in circulating metabolites concentrations associated with AST-120 treatment in advanced CKD patients. The box-plot reveals the median, the 25th, and the 75th percentile. Wilcoxon rank sum test was used to detect differences among groups. * *p* < 0.05. CKD, chronic kidney disease; IS, indoxyl sulfate; pCS, *p* cresyl-sulfate.

Changes of the host-microbe-derived metabolites in CKD patients receiving AST-120, including saturated fatty acids (11 metabolites, Table S1), bile acids (41 metabolites, Table S2), and uremic toxins (IS and pCS) were examined. The concentrations of three fatty acids (acetic acid, octanoic acid, heptanoic acid) were significantly different among three groups. The CKD group exhibited lower serum acetic acid (*p* = 0.004) and decanoic acid (*p* = 0.032) levels and higher serum heptanoic acid (*p* = 0.002) and octanoic acid levels (*p* = 0.074) compared to the non-CKD group. The CKD+AST group restored these abnormalities by increasing serum acetic acid (2.29 ± 0.82 vs. 1.65 ± 0.41 mg/L, *p* = 0.034) and decanoic acid levels (0.27 ± 0.16 vs. 0.23 ± 0.14 mg/L, *p* = 0.633) compared to the CKD patients not on AST-120. The serum concentrations of heptanoic acid were lowest in the non-CKD group, followed by the CKD+AST group and it peaked in the CKD group (0.07 ± 0.10, 0.31 ± 0.48 and 0.69 ± 0.87 mg/L, respectively, *p* < 0.001). The serum concentration of octanoic acid was highest in the CKD+AST-120 groups compared to the CKD and non-CKD groups (0.26 ± 0.2 vs. 0.16 ± 0.15 mg/L vs. 0.15 ± 0.06, *p* = 0.028). Although trends of reductions of total pCS and free pCS were found in the CKD+AST groups as compared to the CKD group; however, the differences were not statistically significant (total pCS, *p* = 0.628; free pCS, *p* = 0.544) (Figure 2). Unexpectedly, serum levels of 41 bile acids did not differ between the CKD and non-CKD groups (Table S2). A slight elevation of serum ursocholic acid levels was noted in the CKD group compared to non-CKD; however, AST-120 administration exacerbated this gap (26.59 ± 34.1 vs. 8.55 ± 8.43 vs. 6.14 ± 5.52 nmol/L in the CKD+AST, CKD and non-CKD groups, respectively, *p* = 0.006, Figure 2, Table S2).

### 3.4. Prediction of Functional Capability of Genes of Gut Microbiota Associated with AST-120

**Figure 3:**
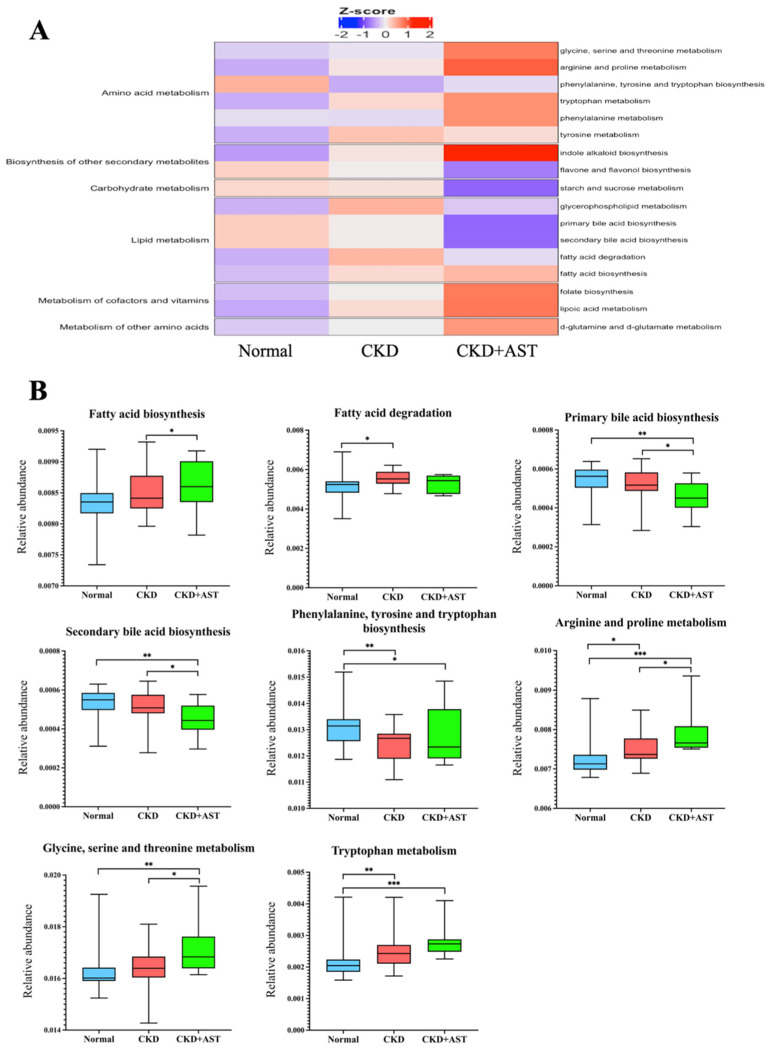
Functional capability of genes of intestinal bacteria among three groups for KEGG metabolism and pathway enrichment were investigated by PICRUSt method. (**A**) Relative abundance of predicted gene function associated with metabolism. (**B**) Specific pathway modules among three groups. Wilcoxon rank sum test was applied to denote variations among groups. * *p* < 0.05; ** *p* < 0.01; *** *p* < 0.001. CKD, chronic kidney disease.

We deduced the functional profile of the genes of gut microbiota by PICRUSt methods [22]. We found that several pathway modules relevant to the metabolism of lipids, amino acids and carbohydrates differed between the CKD+AST and CKD groups (Figure 3A), in addition to the variations of microbial architecture mentioned above. We found that microbial genes related to the fatty acid biosynthesis, arginine and proline metabolism, and glycine, serine and threonine metabolism were differentially enriched; and those genes markers associated with primary and secondary bile acid biosynthesis were depleted in the CKD+AST group compared to the CKD group (Figure 3B). We found an alteration of serum levels of several gut-producing fatty acids among groups. Notably, the changes of genetic markers assigned to the degradation and biosynthesis of fatty acids have corresponded to the variations of these serum fatty acid levels, such as acetic acid and octanoic acid (Figure 2). Although changes of serum levels of IS and pCS associated with the usage of AST-120 were not notorious; however, functional genes associated with phenylalanine, tyrosine and tryptophan biosynthesis, and tryptophan metabolism did differ among CKD+AST-120, CKD group and normal controls (Figure 3B). On the whole, our results denoted significant compositional and functional variations of intestinal microbiome associated with administration of AST-120 in advanced CKD patients. These findings indicate a possible host–microbe–metabolite interaction associated with AST-120 treatment in advanced CKD patients.

## 4. Discussion

The strong absorptive effect of AST-120 has provided insight into the possible “intestinal dialysis intervention” in uremic patients [7,25]; however, the therapeutic effects of AST-120 in retarding renal progression remain controversial. Explorations of biological effects associated with oral absorbents need to be clarified [8,11,26,27]. The present study demonstrated significant changes in the composition of gut microbiota and their functional shift in the strong association with circulating metabolites in advanced CKD patients receiving AST-120. The CKD+AST group had significant increases in the relative abundances of fatty acid-producing bacteria, which were associated with enrichments of the functional module of biosynthesis of fatty acids. Consequently, the CKD+AST group had higher serum levels of acetic acid and octanoic acid. Moreover, the abundances of microorganisms responsible for inflammation-related gastrointestinal diseases, metabolic disorders (family *Erysipelotrichaceae*) [28], and uremic toxins (genus *Coprococcus_3*) [29] were decreased in CKD+AST group. Here we proposed a novel signature involving fatty acids metabolism of the gut microbiota in advanced CKD patients receiving AST-120 therapy. This knowledge provides comprehension to the possible pleotropic effect of AST-120, not merely as an oral adsorbent but also as a potential modifier of gut dysbiosis, to treating renal patients.

Traditionally, AST-120 can ameliorate renal progression by absorption of uremic toxins precursors in the gut and can also interfere with the oxygen sensor system, improving hemoglobin concentrations in CKD patients [3,5,11]. Animal studies revealed that AST-120 may modulate the gut environment and microbiota composition [12,13,30]. Sato et al. have reported that at least 23 gut microbes were significantly changed by renal failure or AST-120 treatment. From these microbes, changes of *Erysipelotrichaceae*, *Clostridium sensu stricto 1*, *Roseburia*, *Faecalibaculum*, *Blautia* and *Desulfovibrio* were correlated with amelioration of fecal *p*-cresol production rather than the adsorptive effects of AST-120. This intestinal modulation may in part explain the different attenuation effects of AST-120 on serum IS and pCS concentrations noted in various studies [12]. Yoshifuji et al. have shown that AST-120 can improve intestinal microbiota, by increasing *Lactobacillus* and *Bacteroides*, and can restore the tight junction function in the colon of uremic rats [13]. The protein expressions of occludin, ZO-1 and claudin-1 were restored as well as the function of toll-like receptors (TLR2/ TLR4) that were regulated by AST-120 [13,14]. For the first time, we demonstrated significant changes of intestinal microbiota architecture in advanced CKD patients receiving AST-120 and the microbial changes were consistent with previous animal studies. We noted differential microbial gene enrichments of phenylalanine, tyrosine and tryptophan biosynthesis and metabolism in patients receiving AST-120. Although we have observed a trend toward reduction in total pCS and free pCS in the circulation of CKD+AST patients; however, the changes did not show a statistical difference in comparison to CKD patients not on AST-120. A small sample size and relatively short duration of AST-120 therapy may in part contribute to these results.

In addition to the uremic toxin-lowering effect, several other lines associated with intestinal microbiota modifications have been reported with administration of AST-120. This compound had anti-cytotoxic effects against *Escherichia coli* and *Pseudomona aeruginosa* by changing gut microbial enzymatic metabolism leading to decreased drug tolerance and virulence [31,32]. AST-120 treatment was associated with a reduced ratio of *Bacteroidetes* to *Firmicutes* (B/F ratio) in the feces of the db/db mice. This variation was correlated with liver weight and steatosis, resulting in suppressed hepatic triglycerides levels [30]. Our findings of a significant increase in fatty acid-producing bacteria (such as *Clostridium_sensu_stricto_1*, *Ruminococcus_2*, *Eubacterium_nodatum* and *Phascolarctobacterium* [33,34]) associated with changes in serum SCFA/MCFA levels and functional gene enrichments of fatty acid biosynthesis in gut microbiota of CKD patients receiving AST-120 administration were novel. SCFAs are carboxylic acids with fewer than six carbon atoms (including acetate, propionate, and butyrate), and MCFAs are fatty acids consisting of 6 to 12 carbon atoms, produced by anaerobic fermentation of indigestible fibers in the intestine. Along with the role as energy-supplying fuel, SCFAs and MCFAs can modulate the metabolism of carbohydrates and lipids [35,36]. These fatty acids, via histone deacetylases inhibition and G-protein-coupled receptors activation can exert various metabolic and immunomodulatory functions and have effects on several cells and organs, including endothelium, immune system, muscle, brain and kidney [37,38,39,40,41]. In contrast to the modulation of a tight junction protein expression and TLR pathway exerted byAST-120, SCFAs strengthen the gut barrier function through the stabilization of the hypoxia-inducible factor, augmentation of barrier oxygenation profile and regulation of epithelial gene expression [42]. SCFAs also intervene on colonocytes energy consumption and regulate mucosa inflammatory cell function; ultimately, promoting intestinal epithelial cells turn-over and restoring the intestinal epithelium [43]. The positive effects of AST-120 on the levels of some SCFA (namely, acetic acid and octanoic acid) may in part explain the anti-inflammatory and renal-protective roles associated with this drug usage [11,13].

The mechanisms by which AST-120 modulates gut microbiota and regulates the production of SCFA remain to be unveiled. AST-120 induces partial restoration of the tight junction proteins [13]. In conjunction with the lowering effects on serum urea and indole-derived toxins, this drug can lead to a lesser extent of urea-derived ammonia and uremic toxins influx into intestinal lumen; subsequently, it can regulate luminal PH, modify substrates for gut microbial metabolism and repair intestinal epithelial barrier damage [12,14]. In addition, AST-120 administration can reduce systemic oxidative stress and inflammation [44,45]. It is unclear if AST-120 also plays a role on the redox reaction of colonocytes and mucosa immune cells. Further trials involving AST-120 with supplementation of different fatty acids may help in elucidating the exact role of oral absorbents in gut dysbiosis and in the preservation of renal function.

Taken together, the results of the present study suggest that administration of AST-120 may ameliorate uremia-induced alteration of the gut microbiome with changes of its byproducts. However, several limitations should be mentioned. Firstly, we included single center, a unique ethnic group with a relatively small number of patients receiving AST-120. Secondly, AST-120 intervention for only three months was short. Other concerns included unavailability of the dose-responsive analysis, un-identification of metabolites in feces to reflect the real intestinal production as well as the lack of oxidative stress markers or cytokines to corroborate the changes in inflammation. However, matching of common confounding baseline factors may minimize the possible bias of our study. To our knowledge, this is the first human study investigating the effects of AST-120 on gut microbiota and their functional adaptions in advanced CKD patients. Further, larger prospective longitudinal multicenter studies with a longer AST-120 intervention period and breakthrough methodologies, such as shotgun metagenomic sequencing and un-targeted metabolomic profiling, may help to decipher the mechanism of AST-120 therapy on intestinal host-microbiome-metabolites synergies in patients with advanced CKD.

## 5. Conclusions

In conclusion, advanced CKD patients exhibited significant gut dysbiosis and variations of serum fatty acids levels. Administration of AST-120 was associated with changes in gut microbiota composition, specifically, increasing fatty acid-producing bacteria. This effect is evidenced by shifts in microbial gene enrichment of fatty acid biosynthesis and by changes in serum SCFA/MCFA levels in advanced CKD patients. Our findings may have not only mechanistic implications in understanding the relationship between AST-120 therapy and fatty acids on the modulation of the gut environment, but also therapeutic insights targeting in the renal-gut axis of renal patients.

## Figures and Tables

**Table 1:** Baseline characteristics of study population (n = 56).

	All Patients, n = 56	Normal, n = 24	CKD, n = 24	CKD+AST, n = 8	*p*
Age, mean (SD)	66.25 ± 7.65	64.04 ± 6.54	68.04 ± 7.31	67.5 ± 10.67	0.691
Male, n (%)	24 (42.9)	12 (50)	10 (41.7)	2 (25)	0.676
Diabetes, n (%)	23 (41.1)	9 (37.5)	12 (50)	2 (25)	0.412
Hypertension, n (%)	44 (78.6)	15 (62.5)	23 (95.8)	6 (75)	0.147
Gout, n (%)	6 (10.7)	0 (0)	4 (16.7)	2 (25)	0.169
Hyperlipidemia, n (%)	25 (44.6)	11 (45.8)	12 (50)	2 (25)	0.133
Systolic pressure, mm Hg	134.20 ± 16.52	128.42 ± 17.02	138.71 ± 14.59	138.57 ± 17.13	0.95
Body mass index kg/m^2^	25.72 ± 4.03	25.87 ± 4.09	25.4 ± 4.1	26.23 ± 4.08	0.482
Blood urea nitrogen, mg/dL	41.73 ± 30.14	14.42 ± 3.62	62.96 ± 22.99	59.96 ± 29.57	0.615
Serum creatinine, mg/dL	3.08 ± 2.61	0.78 ± 0.19	4.51 ± 1.95	5.68 ± 2.83	0.133
Estimated GFR, mL/min/m^2^	52.16 ± 61.07	102.7 ± 63.94	13.94 ± 7.09	15.24 ± 19.48	0.335
Hemoglobin, g/dL	11.15 ± 2.43	13.5 ± 1.18	9.35 ± 1.37	9.54 ± 1.71	0.934
Serum albumin, mg/dL	4.3 ± 0.53	4.55 ± 0.24	4.06 ± 0.67	4.28 ± 0.34	0.904
Serum calcium, mg/dL	8.94 ± 0.67	9.29 ± 0.36	8.7 ± 0.77	8.64 ± 0.62	0.454
Serum phosphate, mg/dL	4.29 ± 1.03	3.79 ± 0.54	4.66 ± 1.09	4.65 ± 1.41	0.6
Serum potassium, mEq/L	4.29 ± 0.58	4.1 ± 0.35	4.41 ± 0.52	4.51 ± 0.97	0.315
Serum uric acid, mg/dL	5.98 ± 1.81	5.64 ± 1.48	6.48± 2.07	5.55 ± 1.79	0.452
Fasting sugar, mg/dL	116.4 ± 36.98	119.42 ± 31.28	116.58 ± 45.98	105.43 ± 16.72	0.58
Total cholesterol, mg/dL	186.25 ± 40.5	192.71 ± 25.05	191.04 ± 46.66	152.5 ± 49.55	0.22
hs-CRP, mg/L	1.46 (2.56)	0.89 (1.21)	2.4 (7.29)	0.8 (2.08)	0.457
Urine protein-creatinine ratio, mg/g	1331.69 (2237.75)	81.5 (122.21)	2144 (1582.46)	1279.5 (701)	0.404
CKD stage, n (%)					0.15
4	8 (14.3)	0	7 (29.2)	1 (12.5)	
5	24 (42.9)	0	17 (70.8)	7 (87.5)	

Legend: Data are shown in mean (SD) or median (interquartile range). *p* value between CKD vs. CKD+AST using median test. Abbreviation: CKD, chronic kidney disease; GFR, glomerular filtration rate; hs-CRP, highly sensitive C reactive protein.

**Table 2:** Change of intestinal microbial genera associated with AST-120 treatment.

Gut Microbiota		RA, Non-CKD	RA, CKD+AST	RA, CKD	*p* *	*p* (CKD5+AST vs. CKD) #	*p* (CKD+AST vs. Non-CKD) #	*p* (CKD vs. Non-CKD) #
Family	Genus							
*Desulfovibrionaceae*	*Bilophila* ↑	0.001321	0.002437	0.001151	0.01777	0.0023	0.0222	0.1221
*Desulfovibrionaceae*	*Desulfovibrio* ↑	0.00131	0.006965	0.002721	0.00005694	0.008106	0.00001035	0.004386
*Synergistaceae*	*Cloacibacillus* ↑	0.00005216	0.001904	0.0001465	0.0003161	0.004336	0.00003474	0.02781
*Synergistaceae*	*Pyramidobacter* ↑	0.00009877	0.002647	0.0003385	0.002609	0.001757	0.0003311	0.246
*Clostridiaceae*	*Hungatella* ↑	0.0004262	0.002574	0.001379	0.0004461	0.01319	0.00006986	0.01236
*Clostridiaceae*	*Clostridium_sensu_stricto_1* ↑	0.00682	0.0283	0.01751	0.0009109	0.04831	0.0002663	0.005404
*Acidaminococcaceae*	*Phascolarctobacterium* ↑	0.001441	0.002211	0.0007402	0.02947	0.004157	0.03693	0.1143
*Eggerthellaceae*	*Eggerthella* ↑	0.0007291	0.001958	0.0009766	0.001362	0.00127	0.0001586	0.2051
*Eubacteriaceae*	*Eubacterium_nodatum* ↑	0.00006437	0.0006392	0.0004018	0.00009063	0.01742	0.00002331	0.002764
*Ruminococcaceae*	*Ruminococcus_2* ↑	0.01591	0.03468	0.01968	0.02802	0.01359	0.003985	0.2649
*Erysipelotrichaceae*	*Erysipelotrichaceae_UCG-003* ↓	0.01026	0.001492	0.007197	0.0001766	0.000575	0.0000165	0.1013
*Erysipelotrichaceae*	*Catenibacterium* ↓	0.01586	0.001242	0.00566	0.0002244	0.01655	0.0000453	0.005829
*Lachnospiraceae*	*Coprococcus_3* ↓	0.005866	0.00245	0.01383	0.006751	0.0009957	0.002919	0.3179
*Lachnospiraceae*	*Lachnospira* ↓	0.002425	0.0005027	0.001978	0.000772	0.02734	0.0001599	0.008844
*Prevotellaceae*	*Prevotella_9* ↓	0.04353	0.001145	0.009813	0.0002952	0.01108	0.00004583	0.01081
*Porphyromonadaceae*	*Barnesiella* ↓	0.003292	0.0004162	0.001276	0.00299	0.03622	0.0005491	0.01896

Legend: Relative abundances (RA) were expressed in %. 1 denotes 100%. Abbreviation: RA, relative abundance; CKD, chronic kidney disease. * Kruskal–Wallis test; # Dunn’s test. ↑ and ↓ denote an increase or decrease in microbial abundance associated AST-120.

## Data Availability

All analytic data were incorporated into the article and the raw data underlying this article will be shared on reasonable request to the corresponding author.

## Supplementary Materials

The following are available online at https://www.mdpi.com/article/10.3390/biomedicines10092234/s1. Table S1: Changes of serum SCFA and MCFA concentration associated with AST-120 (Mean ± SD); Table S2: Changes of serum bile acids concentration associated with AST-120 (Mean ± SD).
