# Supplementary material for: Oral Absorbent AST-120 Is Associated with Compositional and Functional Adaptations of Gut Microbiota and Modification of Serum Short and Medium-Chain Fatty Acids in Advanced CKD Patients
Source: Biomedicines. 2022 Sep 8;10(9):2234. doi: 10.3390/biomedicines10092234 (PMC9496242; doi:10.3390/biomedicines10092234)
Supplement: Supplementary file 1 [file biomedicines-10-02234-s001.zip › biomedicines-1779703-supplementary.pdf]

Table S1. Changes of serum SCFA and MCFA concentration associated with AST-120 (Mean ± SD).

Fatty acids (mg/L)	Molecular formula	Non-CKD	CKD + AST	CKD	*p**	*p*(CKD + AST vs. CKD)#	*p*(CKD + AST vs. non-CKD)#	*p*(CKD vs. non-CKD)#
Acetic acid	C2H4O2	2.06 ± 0.39	2.29 ± 0.82	1.65 ± 0.41	0.011	0.034	0.671	0.004
Propionic acid	C3H6O2	1.15 ± 0.24	0.95 ± 0.52	0.92 ± 0.33	0.115	0.838	0.313	0.029
Isobutyric acid	C4H8O2	0.3 ± 0.08	0.57 ± 0.66	0.75 ± 1.05	0.809	0.891	0.915	0.423
Butyric acid	C4H8O2	1.66 ± 0.47	1.49 ± 1.05	1.33 ± 0.71	0.528	0.453	0.958	0.274
Isovaleric acid	C5H10O2	0.05 ± 0.01	0.06 ± 0.04	0.05 ± 0.02	0.499	0.413	0.21	0.77
Valeric acid	C5H10O2	0.12 ± 0.03	0.13 ± 0.05	0.11 ± 0.02	0.247	0.375	0.873	0.087
Hexanoic aicd	C6H12O2	0.11 ± 0.03	0.18 ± 0.09	0.15 ± 0.14	0.115	0.088	0.137	0.19
Heptanoic acid	C7H14O2	0.07 ± 0.1	0.31 ± 0.48	0.69 ± 0.87	< 0.001	0.682	0.001	0.002

Octanoic acid	C8H16O2	0.15 ± 0.06	0.26 ± 0.2	0.16 ± 0.15	0.028	0.024	0.089	0.074
Nonanoic acid	C9H18O2	0.1 ± 0.02	0.52 ± 0.83	0.73 ± 1.32	0.347	0.246	0.313	0.325
Decanoic acid	C10H20O2	0.32 ± 0.12	0.27 ± 0.16	0.23 ± 0.14	0.1	0.633	0.288	0.032

Abbreviation: SCFA, short-chain fatty acids; MCFA, medium-chain fatty acids; CKD, chronic kidney diseases. *p value among three groups by using Kruskal-Wallis test; #p value between two groups by using Median test.

Table S2. Changes of serum bile acids concentration associated with AST-120 (Mean ± SD).

Bile acids (mM)	Molecular formula	Non-CKD	CKD + AST	CKD	*p**	*p*(CKD + AST vs. CKD)#	*p*(CKD + AST vs. non-CKD)#	*p*(CKD vs. non-CKD)#
12-Dehydrocholic acid	C24H38O5	9.78 ± 0	11.24 ± 4.15	10.47 ± 2.61	0.344	0.632	0.123	0.244
12-Ketolithocholic acid	C24H38O4	9.78 ± 10.19	9.03 ± 7.82	12.43 ± 16.24	0.8	0.443	0.665	0.896
23-Nordeoxycholic acid	C23H38O4	2.22 ± 1.79	17.16 ± 37.64	2.76 ± 2	0.124	0.277	0.047	0.255
3-Dehydrocholic acid	C24H38O5	3.58 ± 4.91	4.38 ± 3.62	2.63 ± 0.65	0.278	0.208	0.173	0.86
7-Ketodeoxycholic acid	C24H38O5	5.84 ± 3.22	20.04 ± 27.48	29.91 ± 87.6	0.056	0.377	0.018	0.092
7-Ketolithocholic acid	C24H38O4	16.48 ± 10.87	35.61 ± 43.9	18.38 ± 16.14	0.564	0.399	0.296	0.966
Allocholic acid	C24H40O5	5.39 ± 1.55	15.41 ± 18.01	6.51 ± 2.8	0.072	0.231	0.024	0.202

Chenodeoxycholic acid	$C_{24}H_{40}O_4$	600.44 ± 787.5	757.9 ± 852.3	408 ± 641.6	0.449	0.453	0.832	0.202
Cholic acid	$C_{24}H_{40}O_5$	215.16 ± 414.45	728.69 ± 1156.38	359.3 ± 804.8	0.382	0.838	0.202	0.291
Deoxycholic acid	$C_{24}H_{40}O_4$	422.61 ± 485.28	605.29 ± 621.64	581.69 ± 996.57	0.44	0.263	0.226	0.97
Glycochenodeoxycholic acid	$C_{26}H_{43}NO_5$	2581.48 ± 6756.84	1346.13 ± 1609.92	1205.63 ± 27447.09	0.099	0.076	0.137	0.166
Glycocholic acid	$C_{26}H_{43}NO_6$	1326.4 ± 4976.21	472.75 ± 896.21	1245.52 ± 3954.95	0.679	0.339	0.524	0.884
Glycodeoxycholic acid	$C_{26}H_{43}NO_5$	612.14 ± 1762.26	821.36 ± 1620.15	1479.7 ± 4964.42	0.356	0.246	0.159	0.827
Glycohyodeoxycholic acid	$C_{26}H_{43}NO_5$	1.76 ± 1.45	3.27 ± 3.45	4.15 ± 7.34	0.442	0.543	0.177	0.607
Glycolithocholic acid	$C_{26}H_{43}NO_4$	9.29 ± 11.4	13.09 ± 14	9.94 ± 16.87	0.327	0.193	0.474	0.248

Glycoursodeoxycholic acid	C26H43NO5	352.15 ± 969.13	304.74 ± 267.2	127.87 ± 125.08	0.182	0.076	0.111	0.942
Glyco-λ-muricholic acid	C26H43NO6	31.26 ± 48.55	28.12 ± 34.6	26.16 ± 48.76	0.87	0.832	0.828	0.601
Hyodeoxycholic acid	C24H40O4	5.44 ± 6.46	19.02 ± 35.26	9.81 ± 16.03	0.256	0.357	0.097	0.466
Isolithocholic acid	C24H40O3	13.81 ± 9.29	12.13 ± 6.68	11.17 ± 3.55	0.762	1	0.611	0.516
Lithocholic acid	C24H40O3	16.04 ± 12.41	14.66 ± 5.22	13.14 ± 7.31	0.596	0.318	0.62	0.515
Taurochenodeoxycholic acid	C26H45NO6S	594.07 ± 1504.58	327.49 ± 507.39	255.47 ± 259.48	0.574	1	0.339	0.423
Taurocholic acid	C26H45NO7S	659.03 ± 2521.56	195.72 ± 384.74	398.78 ± 1066	0.492	0.679	0.563	0.238

Taurodeoxycholic acid	C26H45NO6S	82.64 ± 214.14	170.15 ± 323.25	152.74 ± 352.11	0.397	0.837	0.389	0.199
Taurolithocholic acid	C26H45NO5S	9.77 ± 0	12.22 ± 4.52	10.47 ± 2.61	0.08	0.251	0.026	0.244
Tauroursodeoxycholic acid	C26H45NO6S	22.58 ± 35.79	24.71 ± 15.98	20.86 ± 15.35	0.099	0.518	0.025	0.194
Ursocholic acid	C24H40O5	6.14 ± 5.52	26.59 ± 34.1	8.55 ± 8.43	0.006	0.057	0.002	0.177
Ursodeoxycholic acid	C24H40O4	123.6 ± 161.65	280.42 ± 373.98	109.8 ± 154.1	0.15	0.101	0.063	0.855
β-Muricholic acid	C24H40O5	2.58 ± 0.56	8.86 ± 18.12	2.8 ± 0.89	0.658	1	0.483	0.38
λ-Muricholic acid	C24H40O5	25.3 ± 13.16	55.86 ± 45.09	29.9 ± 13.44	0.075	0.184	0.029	0.225
α-Muricholic acid	C24H40O5	UD	UD	UD	-	-	-	-

6,7-Diketolithocholic acid	C24H36O5	UD	UD	UD	-	-	-	-
7,12-Diketolithocholic acid	C24H36O5	UD	UD	UD	-	-	-	-
Allolithocholic acid	C24H40O3	UD	UD	UD	-	-	-	-
Apocholic acid	C24H38O4	UD	UD	UD	-	-	-	-
Dehydrocholic acid	C24H34O5	UD	UD	UD	-	-	-	-
Dehydrolithocholic acid	C24H38O3	UD	UD	UD	-	-	-	-
Isodeoxycholic acid	C24H40O4	UD	UD	UD	-	-	-	-
Tauro α-Muricholic acid	C26H45NO7S	UD	UD	UD	-	-	-	-

Tauro β-Muricholic acid	C26H45NO7S	UD	UD	UD	-	-	-	-
Taurohyodeoxycholic acid	C26H45NO6S	UD	UD	UD	-	-	-	-
Glycodehydrocholic acid	C26H37NO6	UD	UD	UD	-	-	-	-

Abbreviation: CKD, chronic kidney disease; UD, undetectable. *p value among three groups by using Kruskal-Wallis test; #p value between two groups by using Median test.
